# Overview of popular cosmeceuticals in dermatology

**DOI:** 10.1002/ski2.340

**Published:** 2024-02-07

**Authors:** Chantalle Crous, Judey Pretorius, Anél Petzer

**Affiliations:** ^1^ Pharmaceutical Chemistry School of Pharmacy and Centre of Excellence for Pharmaceutical Sciences North‐West University Potchefstroom South Africa; ^2^ Biomedical Emporium Pretoria South Africa

## Abstract

The eternal pursuit to prevent ageing and maintain a youthful appearance has resulted in a rapidly expanding cosmeceutical industry. Cosmeceutical products, particularly of natural origin, are in high demand due to claims of efficacy for signs of ageing and other skin conditions. Consumers often include cosmeceutical products in their skin care regime as they are readily available, and a more affordable option compared to prescription products. However, many cosmeceutical ingredients lack clinical evidence regarding their efficacy and safety as these products are not regulated by the U.S. Food and Drug Administration. This review provides a brief overview of several popular cosmeceutical ingredients with regards to their potential indications, targets and mechanisms of action.

## Graphical abstract

Cosmeceutical products are often included in skin care regimes due to availability and affordability. However, many cosmeceutical ingredients lack clinical evidence. This article provides an overview of several popular cosmeceutical ingredients with regards to potential indications, targets and mechanisms of action.

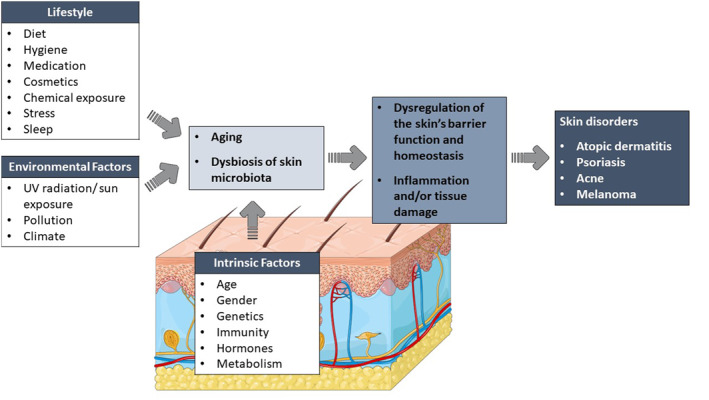

**What is already known about this topic?**

- Various cosmeceutical ingredients are incorporated in skincare products for treatment of several skin conditions.

**What does this study add?**

- This review highlights the potential indications, targets, and mechanisms of action of several popular cosmeceuticals.

## 1 INTRODUCTION

**FIGURE 1:**
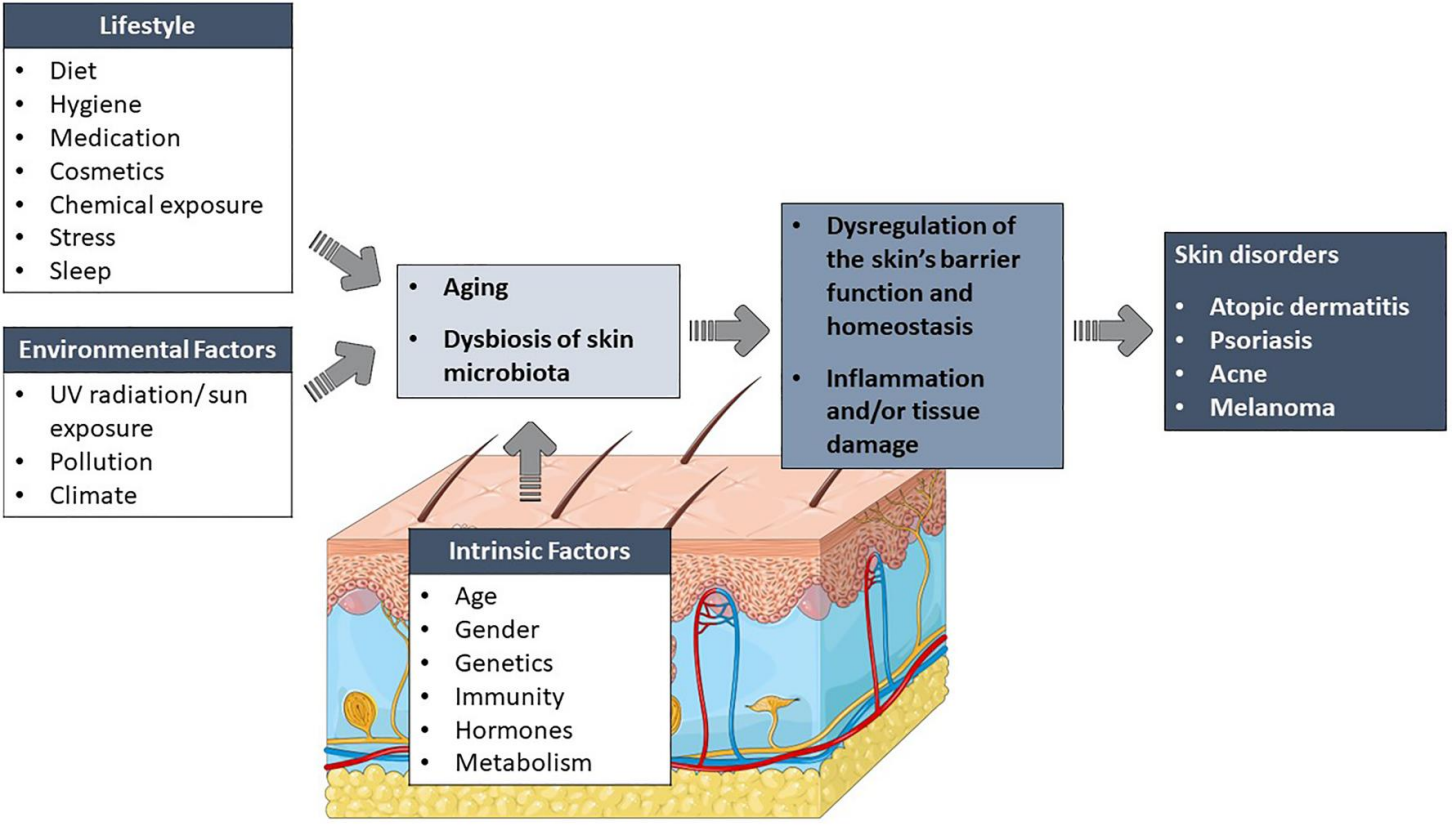
The effects of various intrinsic and extrinsic factors on the skin.

The skin is subjected to various intrinsic and extrinsic factors resulting in ageing (Figure 1). Intrinsic or chronological ageing refers to the natural skin ageing process and is characterised by an accumulation of senescent skin cells with reduced proliferative ability, that are resistant to apoptosis, and release pro‐inflammatory mediators.^1^ These changes are responsible for the classic signs of ageing – wrinkle formation, coarse skin texture, dyspigmentation, dryness and reduced elasticity.^1^ Extrinsic factors such as environmental pollution, ultraviolet (UV) radiation exposure, stress, diet and sleep deprivation, accelerate the ageing process. Both intrinsic and extrinsic factors also significantly affect the skin's natural microbiota which consists of various micro‐organisms such as bacteria, viruses, fungi and micro‐eukaryotes.^2^, ^3^ The host‐microbiota relationship is mutually beneficial – while the host provides a protective, nutrient‐rich environment, the microbiota plays a crucial role in maintaining skin homoeostasis and barrier function.^4^, ^5^, ^6^ In addition to providing a physical barrier against pathogenic microbes via various antagonistic mechanisms, skin microbiota also enhances the host's immune response.^4^, ^6^ However, disruption of the skin's ecosystem (i.e. dysbiosis) can mediate inflammation and/or tissue damage which has been linked to a variety of skin disorders (e.g. atopic dermatitis, psoriasis, acne, melanoma and chronic wounds).^5^, ^7^

The use of cosmeceuticals, especially for anti‐ageing, is a rapidly growing field of interest. Cosmeceuticals refer to bioactive compounds that demonstrate both cosmetic and therapeutic benefits.^8^ These bioactive compounds are generally of natural origin (e.g. plants, animals or micro‐organisms), although synthetic derivatives are also used, and display various beneficial properties including antioxidant, anti‐inflammatory and antimicrobial action.^8^

The current review provides a brief overview of several cosmeceutical ingredients commonly used by consumers and skin care professionals (e.g. aesthetic doctors, dermatologists, plastic surgeons, somatologists and beauty therapists). Readers are also referred to other review articles focussing on specific cosmeceuticals for a more detailed discussion. Databases (including PubMed, Science Direct, Web of Science and Google Scholar) were searched for appropriate publications. The following keyword were used: cosmeceuticals, natural products, ageing, skincare, skin conditions and treatments. No date restrictions were used during the search. Only English, full‐text articles considered to be relevant were reviewed. The reference list of identified papers was used to identify any further papers of interest.

## 2 VITAMINS

Vitamins play an important role in maintaining skin health and treating skin disorders, thus they are often incorporated in various cosmeceutical products. In this section a brief overview will be given on vitamins A, B_3_, B_5_, C, D and E. Readers are referred to recent review articles for a more detailed discussion on vitamin studies and the levels of evidence for their role in treatment of skin disorders.^9^, ^10^

### 2.1 Retinoids

Retinoids are fat‐soluble vitamins consisting of vitamin A (also known as retinol), its natural derivatives (retinaldehyde or retinal, retinoic acid and retinyl esters) and various synthetic derivatives.^9^ Retinol and retinyl esters are obtained through diet and converted in vivo to the biologically active form, retinoic acid.^9^ Retinoids are involved in immune function, embryonic development, vision, cellular differentiation, communication and apoptosis.^11^

Due to their lipophilicity, retinoids are transported intracellularly via cytosolic retinol binding protein (CRBP I and II) or cytosolic retinoic acid‐binding protein (CRABP I and II).^10^ Once inside the nucleus, retinoids' effects are mediated through two types of nuclear receptor families, retinoic acid receptors (RARs, consisting of RAR‐α, ‐β and ‐γ) and retinoid X receptors (RXRs, consisting of RXR‐α, ‐β and ‐γ), which regulate gene transcription.^11^ Retinoids have different affinities for specific receptor subtypes which affect their potency and tolerability (Table 1).^11^

**TABLE 1:** Topical retinoids as cosmeceutical ingredients.^11^

Retinoid	Receptor specificity	Indications	Mechanism of action
Retinyl esters (e.g. retinyl acetate and palmitate)	Converted to retinoic acid with RAR‐α, ‐β, ‐γ activity	Anti‐ageing	**Acne vulgaris:** Stimulates epidermal cell turnover and detachment of cornified cells. This reduces mature comedones and inhibits new comedone formation.
Retinol		Anti‐ageing	
Retinaldehyde (Retinal)		Anti‐ageing	
Tretinoin (All‐*trans* retinoic acid)	RAR‐α, ‐β, ‐γ	Acne vulgaris, Photoaging	**Anti‐ageing/Photoaging:** Reduces MMP activity to prevent collagen degradation. Stimulates collagen biosynthesis.
		Off‐label uses: Hyperpigmentation	
Tazarotene	RAR‐β, ‐γ	Acne vulgaris, Plaque psoriasis	**Plaque psoriasis:** Inhibits keratinocyte hyperproliferation and inflammatory mediators. Stimulates keratinocyte differentiation.
Adapalene	RAR‐β, ‐γ	Acne vulgaris	
		Off‐label uses: Hyperpigmentation, Photoaging	
Trifarotene	RAR‐γ	Acne vulgaris	
Seletinoid G	RAR‐γ	Photoaging	**Hyperpigmentation:** Inhibits melanogenesis via tyrosinase inhibition or modulating tyrosinase transcription.
			Inhibits melanin transfer from melanocytes to keratinocytes.
			Stimulates epidermal cell turnover thus removing melanin‐laden keratinocytes.
Alitretinoin (9‐*cis*‐retinoic acid)	RAR‐α, ‐β, ‐γ	Kaposi's sarcoma	Inhibits the cell cycle resulting in reduced neoplastic cell proliferation and increased apoptosis.
	RXR‐α, ‐β, ‐γ		
Bexarotene	RXR‐ α	Cutaneous T‐cell lymphoma	Inhibits the cell cycle resulting in reduced T‐cell proliferation and increased apoptosis.

Abbreviations: MMP, matrix metalloproteinase; RAR, retinoic acid receptor; RXR, retinoid X receptor.

Topical retinoids are primarily indicated for the treatment of psoriasis, acne vulgaris, photoaging, T‐cell lymphoma and Kaposi's sarcoma; however, these compounds also have various off‐label uses for treatment of other dermatological conditions (Table 1).^9^, ^11^ Based on their structural properties and receptor subtype selectivity, retinoids are divided into four generations^9^, ^10^, ^11^:

- First generation: Natural retinoids with non‐selective receptor subtypes. These include retinol, retinaldehyde, all‐*trans* retinoic acid (tretinoin), 9‐*cis*‐retinoic acid (alitretinoin) and 13‐*cis*‐retinoic acid (isotretinoin – oral retinoid).
- Second generation: Monoaromatic, oral retinoids such as etretinate and acitretin. Currently, no topical second‐generation retinoids are available.
- Third generation: Polyaromatic retinoids with more selectivity towards specific receptor subtypes than previous generations, such as adapalene, tazarotene and bexarotene.
- Fourth generation: Trifarotene and seletinoid G with receptor selectivity for epidermal RAR‐γ.

Retinoid formulations are classified as either over the counter (cosmetic) or prescription retinoids. Cosmetic retinoids consist of the precursors to retinoic acid (e.g. retinyl esters, retinol and retinaldehyde) that require enzymatic conversion in the skin.^11^ These retinoids are stable in topical formulations; however, they are less effective.^11^ Prescription retinoids include tretinoin, tazarotene, adapalene (0.1% adapalene formulation is also available over the counter) and trifarotene.^11^ Along with higher efficacy, prescription retinoids also have more adverse effects including skin irritation, dryness, erythema, pruritis and burning.^11^ The use of topical retinoid products is contraindicated during pregnancy and breastfeeding, and for individuals with retinoid hypersensitivity and sensitive skin (e.g. dermatitis).^11^

### 2.2 B vitamins

Niacinamide or nicotinamide (a vitamin B_3_ derivative) is a precursor for nicotinamide adenine dinucleotide coenzymes (NAD^+^, NADH, NADP^+^ and NADPH) involved in cellular processes such as adenosine triphosphate (ATP) production, DNA repair, signalling and gene expression.^12^ Topical niacinamide is often used for treatment of hyperpigmentation, photoaging, acne vulgaris and inflammatory skin conditions such as rosacea (Table 2).^10^, ^12^, ^13^ Commercial niacinamide products are generally formulated at concentrations up to 5%, although certain products contain up to 10%.^12^ Depending on skin sensitivity, topical niacinamide (<5%) is well tolerated with no or few side‐effects (e.g. mild skin irritation, erythema and pruritis).^12^ Clinical efficacy of various niacinamide formulations (monotherapy or combination regimes) was discussed in detail in a recent review.^12^

**TABLE 2:** Vitamin analogues as cosmeceutical ingredients.

Vitamin/derivatives	Potential indications	Mechanism of action/Target
**Vitamin B**_**3**_		
Niacinamide	Hyperpigmentation, Photoaging, Acne vulgaris, Inflammatory skin conditions (e.g. rosacea)	**Anti‐inflammatory** ^10^, ^12^, ^13^:Inhibits inflammatory mediators (PARP‐1, TNF‐α, IL‐8, IL‐10, NF‐ĸB and AP‐1).
		**Photoprotective** ^10^, ^12^, ^13^:Stimulates ATP production and DNA repair.
		**Whitening effect** ^10^, ^12^, ^13^:Inhibits melanosome transfer from melanocytes to keratinocytes. Inhibits factors involved in melanin production (e.g. MITF, TRP‐1, TRP‐2 and PMEL17).
		**Anti‐ageing** ^10^, ^12^, ^13^:Co‐factor to enzymes involved in production of ceramides, keratin, collagen and elastin. Reduces transepidermal water loss and prevents fine lines and wrinkles.
**Vitamin B**_**5**_		
Dexpanthenol	Xerosis, Atopic dermatitis, Diaper rash, Wound healing, Scar management, Alopecia	**Moisturising** ^14^:Hygroscopic properties ‐ promotes water retention and reduces transepidermal water loss.
		**Wound healing** ^16^:Anti‐inflammatory effects. Stimulates fibroblast proliferation and epithelialisation. Upregulates genes involved in wound healing (e.g. IL‐6, IL‐1β, CXCL1, CCL18, CYP1B1 and KRTAP 4‐2 or KAP 4‐2).
		**Scar management** ^14^:Hydrates skin and reduces transepidermal water loss. Strengthens the skin barrier in scar tissue.
		**Hair growth** ^19^:Stimulates proliferation of hair follicle cells and reduces cell senescence and apoptosis. Lengthens anagen phase by reducing TGF‐β expression. Upregulates VEGF expression which promotes blood circulation to hair follicles.
**Vitamin C**		
L‐ascorbic acid Magnesium ascorbyl phosphate Sodium L‐Ascorbyl‐2‐phosphate Disodium isostearyl 2‐O‐L‐ascorbyl phosphate Ascorbic 2‐phosphate 6‐palmitate Ascorbyl tetraisopalmitate Tetrahexyldecyl ascorbate Ascorbyl 2‐glucoside Ascorbyl 6‐palmitate 3‐O‐Ethyl ascorbic acid	Hyperpigmentation, Photoaging, Anti‐ageing (reduction in wrinkles), Rosacea, Acne vulgaris	**Antipigmentary** ^10^:Inhibits tyrosinase by interacting with the copper ions of the active site, thus reducing melanogenesis. Promotes conversion of melanin to leucomelanin (colourless pigment).
		**Antioxidant** ^21^:Neutralises free radicals from UV exposure and environmental pollution, thus preventing oxidative damage to the skin. Regeneration of oxidised Vitamin E.
		**Photoprotective** ^9^, ^21^:Inhibits AP‐1 upregulation caused by UV exposure. This reduces MMP synthesis and subsequent collagen degradation. Inhibits elastin biosynthesis which is upregulated during UV exposure (in vitro studies). Prevents decrease in CD1a‐expressing Langerhans cells, thus reducing UV‐induced immunosuppression. Reduces the formation of thymine dimers, a potential factor for photocarcinogenesis.
		**Anti‐ageing** ^12^, ^36^:Involved in multiple stages of collagen biosynthesis. Stimulates transcription of procollagen I and III genes. Co‐factor of hydroxylase enzymes involved in stabilisation of collagen molecules. Promotes barrier functions – keratinocyte differentiation, barrier lipid biosynthesis and organisation – prevents transepidermal water loss.
		**Anti‐inflammatory** ^36^:Inhibits NF‐ĸB and subsequent activation of pro‐inflammatory cytokines (e.g. IL‐1, IL‐6, IL‐8 and TNFα).
**Vitamin D**		
Calcipotriene (also known as calcipotriol)	Psoriasis	Inhibits keratinocyte hyperproliferation and pro‐inflammatory cytokines (IL‐2, IL‐6 and IFN‐γ) upregulated in psoriatic lesions.^25^
Calcitriol	Actinic keratosis	Stimulates TSLP expression by epithelial cells which induces an antitumour T‐cell response.^25^
Tacalcitol	Morphoea/localised scleroderma	Inhibits fibroblast proliferation and T‐cell activation. Reduces TGF‐β stimulation of fibroblasts, which inhibits myofibroblast differentiation and collagen synthesis.^25^
Maxacalcitol		
**Vitamin E**		
α‐Tocopherol Tocopheryl acetate Tocopheryl succinate Tocopheryl linoleate Tocopheryl nicotinate Tocopheryl glucoside Tocopheryl phosphate	Hyperpigmentation, Photoaging, UV protection (sunscreens), Anti‐ageing, Melanoma, Inflammatory skin disorders (e.g. psoriasis and atopic dermatitis)	**Antipigmentary** ^32^:Downregulation of TYR, TYRP1 and TYRP2 gene expression. Inhibits tyrosinase activity, thus reducing melanogenesis.
		**Antioxidant** ^33^:Neutralises lipid peroxyl free radicals to protect cell membranes from lipid peroxidation.
		**Photoprotective** ^33^, ^37^:Prevents IL‐8 expression and AP‐1 activation during UV exposure (reduces erythema and oedema of sunburn). Reduces the formation of cyclobutane pyrimidine dimer formation, a potential factor for photocarcinogenesis.
		**Anti‐inflammatory** ^33^, ^38^:Inhibits the production of prostaglandin and pro‐inflammatory cytokines, COX‐2 and NOX. Modulates protein kinase C and PI3K signalling pathways.
		**Anti‐ageing** ^33^, ^39^, ^40^:Reduces collagenase expression (via protein kinase C pathway modulation) which is upregulated in ageing skin. This reduces collagen degradation. Upregulation of collagen gene expression and downregulation of MMP gene expression – stimulating collagen synthesis and inhibiting collagen degradation. Improves skin barrier function to prevent transepidermal water loss, thus increasing skin hydration.
		**Anti‐tumour** ^33^, ^41^:Stimulates tumour cell apoptosis, causes cell cycle arrest and inhibits VEGF‐mediated angiogenesis which slows down melanoma growth.

Abbreviations: AP, activator protein; ATP, adenosine triphosphate; CCL, chemokine (C‐C motif) ligand; COX, cyclooxygenase; CXCL, chemokine (C‐X‐C motif) ligand; CYP, cytochrome P450; IFN‐γ, interferon gamma; IL, interleukin; KRTAP or KAP, keratin‐associated protein; MITF, microphthalmia‐associated transcription factor; MMP, matrix metalloproteinase; NF‐κB, nuclear factor kappa B; NOX, nicotinamide adenine dinucleotide phosphate (NADPH) oxidase; PARP, poly (ADP‐ribose) polymerase; PI3K, phosphatidylinositol 3‐kinase; PMEL, premelanosome protein; TGF‐β, transforming growth factor beta; TNF‐α, tumor necrosis factor alpha; TRP or TYRP, tyrosinase‐related protein; TSLP, thymic stromal lymphopoietin; TYR, tyrosinase; UV, ultraviolet; VEGF, vascular endothelial growth factor.

Vitamin B_5_ or pantothenic acid is a water‐soluble vitamin required for the biosynthesis of coenzyme A.^14^ Coenzyme A is an essential co‐factor involved in various biological processes including energy production and fatty acid synthesis. Provitamin B_5_ (D‐panthenol or dexpanthenol), a precursor of pantothenic acid, is often used in skin‐ and haircare products.^15^ Topically applied dexpanthenol is well absorbed through the skin and demonstrates moisturising, barrier‐improving and anti‐inflammatory properties.^15^ Dexpanthenol‐containing products are often used to treat and prevent xerosis, pruritis and skin irritation in conditions such as atopic dermatitis and diaper rash (Table 2).^14^, ^15^ In vitro and in vivo studies have also demonstrated promising results for topical dexpanthenol in wound healing and scar management.^14^, ^16^ The use of dexpanthenol in haircare products may stimulate hair growth in patients with alopecia.^17^, ^18^, ^19^

### 2.3 Vitamin C

Vitamin C or ascorbic acid is a water‐soluble antioxidant obtained through diet and supplementation. L‐ascorbic acid is the biologically active form of vitamin C and acts as co‐factor for various enzymes regulating immune function, iron metabolism, and the biosynthesis of collagen, neurotransmitters, hormones and skin barrier lipids.^12^ Vitamin C is a popular cosmeceutical ingredient incorporated in numerous skincare products for hyperpigmentation, photodamage, anti‐ageing and acne vulgaris (Table 2).^20^

Due to instability and poor skin penetration of L‐ascorbic acid (hydrophilic, charged molecule at neutral pH), several esterified vitamin C derivatives have been developed for topical formulations.^21^, ^22^ These derivates are stable at neutral pH and lipophilic which enhances transdermal delivery.^21^ For a more detailed discussion on the different vitamin C derivatives, their efficacies and limitations, readers are referred to a recent review by Enescu and co‐workers.^16^ Topical formulations generally contain 10%–20% vitamin C, which is well tolerated for long‐term, daily use.^21^ However, individuals with sensitive skin have reported skin irritation, erythema, pruritis and dryness with high concentrations of vitamin C.^10^

### 2.4 Vitamin D

Vitamin D is a prohormone obtained through diet and produced by epidermal keratinocytes when exposed to UV radiation.^9^ Vitamin D is converted to its active form (1,25‐dihydroxycholecalciferol or calcitriol) in the liver and kidneys and regulates calcium‐phosphorous homoeostasis, immune response, cell growth and differentiation.^9^ The effects of calcitriol and other vitamin D analogues are mediated through vitamin D receptors, inducing either rapid signalling effects or gene transcription.^23^

Vitamin D also regulates various biological processes in the skin including keratinocyte proliferation, differentiation and apoptosis, and maintaining the skin barrier and immune response.^9^ Due to its vital role, vitamin D has been implicated in the pathophysiology of various skin disorders (Table 2). Psoriasis, an inflammatory skin condition, is characterised by keratinocyte hyperproliferation, aberrant keratinocyte differentiation and upregulation of pro‐inflammatory mediators.^24^ Topical vitamin D analogues, used either as monotherapy or in combination with topical corticosteroids, have demonstrated efficacy in the treatment of psoriasis.^25^ Topical calcitriol ointment (3 mcg/g), calcipotriene foam (0.005%) and calcipotriene/betamethasone dipropionate (0.005%/0.064%) was approved by the U.S. Food and Drug Administration (FDA) for mild to moderate psoriasis.^26^, ^27^, ^28^ The most common adverse effects of these formulations include skin irritation, pruritis, burning and erythema.^26^, ^27^, ^28^

Topical vitamin D analogues may also have potential for treatment of other skin conditions. Actinic keratosis is a precancerous skin condition caused by chronic UV exposure. Vitamin D analogues stimulate epithelial expression of thymic stromal lymphopoietin (TSLP) which induces a T‐cell‐mediated antitumour response.^25^ Topical calcipotriene ointment (0.005%) used in combination with the standard actinic keratosis treatment, 5‐fluoruracil cream (5%), demonstrated a synergistic antitumour effect in actinic keratosis patients, with a mean reduction of 87.8% compared to the 26.3% for the 5‐fluoruracil control group.^29^ Morphoea or localised scleroderma is characterised by inflammation, fibrosis and dyspigmentation of the skin. Topical calcipotriene ointment (0.005%) as monotherapy or in combination with low‐dose ultraviolet A1 (UVA1) phototherapy, significantly reduced morphoea in children and adults.^30^, ^31^ The efficacy of calcipotriene could possibly be attributed to the inhibition of fibroblast hyperproliferation and T‐cell activity. Calcipotriene can also reduce fibroblast’ sensitivity to transforming growth factor beta (TGF‐β) stimulation, resulting in inhibition of myofibroblast differentiation and excessive collagen production.^25^

### 2.5 Vitamin E

Vitamin E is a family of lipid‐soluble vitamins consisting of two groups, tocopherols and tocotrienols, with four isomers (α‐, β‐, γ‐, δ‐) in each group.^32^ α‐Tocopherol is the most abundant isoform in human tissues and skin, followed by γ‐tocopherol. All vitamin E isomers are potent antioxidants responsible for scavenging lipid peroxyl free radicals and thus protecting cell membranes from lipid peroxidation.^33^ Due to its potent antioxidant and anti‐inflammatory activity, vitamin E is another popular ingredient used in commercial products for hyperpigmentation, UV protection, anti‐ageing and inflammatory skin disorders (e.g. psoriasis and atopic dermatitis) (Table 2).^32^, ^33^ Tocopherol esters (e.g. tocopheryl acetate, ‐glucoside and ‐phosphate) are often used in topical products, which is converted to biologically active tocopherol in vivo.^32^, ^34^ These esters are more stable and less prone to oxidation than α‐tocopherol; however, the extent of conversion in the skin affects bioavailability.^32^, ^33^ In vivo, tocopherol's antioxidant capacity is regenerated via hydrophilic co‐antioxidants such as vitamin C and glutathione, thus topical products often contain a combination of antioxidants.^35^ Combination formulations often demonstrate better efficacy compared to single antioxidant formulations. Tocopherol concentration in topical formulations is generally between 1% and 5%, which is well‐tolerated.^35^ Adverse effects of these products are rare, with only few cases of mild skin irritation reported.^35^

## 3 COENZYME Q10

Coenzyme Q10 is an endogenous, lipophilic co‐factor present in biological membranes in both its oxidised (ubiquinone) and reduced (ubiquinol) forms.^42^ It plays an essential role in the electron transport chain during energy production.^9^, ^42^ Ubiquinol acts as antioxidant which protects cell membranes against lipid peroxidation and regenerates other antioxidants (e.g. L‐ascorbic acid and α‐tocopherol).^9^, ^42^ Anti‐ageing products often include coenzyme Q10 in their formulations due to these properties. In addition, in vitro studies have demonstrated the potential anti‐inflammatory, photoprotective and skin whitening activity of coenzyme Q10.^43^ Coenzyme Q10 reduced interleukin (IL)‐1α, reactive oxygen species (ROS) and matrix metalloproteinase (MMP)‐1 production in UV‐irradiated cells, and enhanced collagen and elastin gene expression.^43^ Coenzyme Q10 also inhibited tyrosinase activity in melanoma cells, thus inhibiting melanogenesis.^43^

## 4 PEPTIDES

Bioactive peptides are protein fragments that facilitate cellular communication for various biological processes including immune response, stress response, homoeostasis and growth.^44^ Both natural and synthetic bioactive peptides are used as cosmeceutical ingredients to treat various skin conditions. Based on their mechanism of action, bioactive peptides are classified as carrier‐, signal‐ and neurotransmitter inhibitor peptides (Table 3).^44^ Carrier peptides facilitate the transportation of co‐factors such as copper and manganese across the skin barrier. Both co‐factors are essential for several enzymatic reactions involved in anti‐ageing and wound healing.^44^ Copper tripeptide‐1 and manganese tripeptide‐1 are examples of carrier peptides that have been used successfully in reducing fine lines, wrinkles and hyperpigmentation associated with photoaging.^45^, ^46^, ^47^, ^48^

**TABLE 3:** Examples of cosmeceutical peptides.

Peptides	Sequence	Indications	Mechanism of action/Target
**Carrier peptides**			
Copper tripeptide‐1	Cu(II) H‐Gly‐His‐Lys‐OH (Cu‐GHK)	Anti‐ageing, Wound healing, Hyperpigmentation	Copper acts as co‐factor for lysyl oxidase involved in collagen and elastin biogenesis.
			Regulates MMP and collagenase activity.
			Anti‐inflammatory and antioxidant properties.
			Stimulates angiogenesis.
			Inhibits melanin synthesis.^45^, ^47^, ^48^
Manganese tripeptide‐1	Mn(II) H‐Gly‐His‐Lys‐OH (Mn‐GHK)	Hyperpigmentation associated with photoaging	Manganese acts as co‐factor for superoxide dismutase, an antioxidant that protects skin against photo‐oxidative damage.^46^
**Signal peptides (Matrikines)**			
Palmitoyl tripeptide‐1 (Biopeptide CL™)	Pal‐Gly‐His‐Lys‐OH (GHK)	Anti‐ageing (reduces wrinkles)	Stimulates collagen and glycosaminoglycan synthesis.^45^
Palmitoyl hexapeptide‐12 (Biopeptide EL™)	Pal‐Val‐Gly‐Val‐Ala‐Pro‐Gly‐OH (VGVAPG)	Anti‐ageing (improves skin firmness and elasticity)	Stimulates production of collagen, elastin, fibronectin and glycosaminoglycans.
			Inhibits IL‐6 to reduce inflammation and ECM degradation.
			Stimulate fibroblast mobility.^45^, ^51^
Palmitoyl tripeptide‐5 (Syn®‐Coll)	Pal‐Lys‐Val‐Lys‐OH (KVK)	Anti‐ageing (improves skin firmness and elasticity),	Stimulates TGF‐β to induce collagen synthesis.
		Hyperpigmentation	Inhibits MMP degradation of collagen.
			Inhibits melanin synthesis.^45^, ^51^
Palmitoyl pentapeptide‐4 (Matrixyl®)	Pal‐Lys‐Thr‐Thr‐Lys‐Ser‐OH (KTTKS)	Anti‐ageing (reduces medium and deep wrinkles)	Stimulates production of ECM proteins for example, collagens (Type I, III and IV), elastin, fibronectin and glycosaminoglycan.^45^, ^51^
Combination of palmitoyl tripeptide‐1 and palmitoyl tetrapeptide‐7 (Matrixyl 3000™)	Pal‐Gly‐His‐Lys‐OH (GHK) and Pal‐Gly‐Gln‐Pro‐Arg‐OH (GQPR)	Anti‐ageing (Reversing skin ageing – deep wrinkles, loss of firmness, and photodamage)	Stimulates production of ECM proteins for example, collagens (Type I, III and IV), elastin, fibronectin and glycosaminoglycan.
			Inhibits IL‐6 to reduce inflammation and ECM degradation.^52^
Palmitoyl tripeptide‐38 (Matrixyl Synthe'6™)	Pal‐Lys‐Met(O_2_)‐Lys‐OH (KXK)	Anti‐ageing (preventing the first signs of ageing – fine lines and surface wrinkles)	Stimulates production of ECM components such as collagens (Type I, III and IV), elastin, fibronectin, glycosaminoglycan, hyaluronic acid and laminin.^52^
Tripeptide‐10 citrulline (Decorinyl®)	H‐Lys‐Asp‐Ile‐Cit‐NH_2_ (KDI‐Cit)	Anti‐ageing (improves skin firmness and elasticity)	Mimics the function of decorin to regulate collagen fibrillogenesis. Targets collagen organisation without affecting collagen synthesis.^53^
Heptapeptide (Perfection Peptide P7™)	Ac‐Asp‐Glu‐Glu‐Thr‐Gly‐Glu‐Phe‐OH (DEETGEF)	Protection against photoaging	Stimulates Nrf2‐dependant antioxidant enzymes to protect cellular DNA against UV damage.^44^
Decapeptide (SA1‐III or KP‐1)	Ac‐Met‐Gly‐Lys‐Val‐Val‐Asn‐Pro‐Thr‐Gln‐Lys (MGKVVNPTQK)	Anti‐ageing (improves skin firmness and elasticity)	Prevents collagen degradation by inhibiting proteases (MMPs and elastase). Does not affect collagen synthesis.^45^, ^54^
Oligopeptide‐68 (β‐WHITE™)	H‐Arg‐Asp‐Gly‐Gln‐Ile‐Leu‐Ser‐Thr‐Trp‐Tyr‐OH (RDGQILSTWY)	Whitening agent for melasma	Inhibits MITF involved in melanin production.^44^
**Neurotransmitter inhibitor peptides**			
Acetyl hexapeptide‐3 (Argireline®)	Ac‐Glu‐Glu‐Met‐Gln‐Arg‐Arg‐NH_2_ (EEMQRR)	Anti‐ageing (reduces wrinkles)	SNAP‐25 protein sequence which prevents SNARE complex formation and subsequent acetylcholine release.^45^, ^51^
Pentapeptide‐3 (Vialox)	H‐Gly‐Pro‐Arg‐Pro‐Ala‐NH_2_ (GPRPA)	Anti‐ageing (reduces wrinkles)	Acetylcholine receptor antagonist derived from snake venom.^45^, ^51^
Pentapeptide‐18 (Leuphasyl®)	H‐Tyr‐Ala‐Gly‐Phe‐Leu‐OH (YAGFL)	Anti‐ageing (reduces wrinkles)	Decreases acetylcholine release in the synaptic cleft.^45^, ^51^
Tripeptide‐3 (Syn®‐Ake)	H‐β‐Ala‐Pro‐Dab‐NH‐benzyl x 2AcOH (dipeptide diaminobutyroyl benzylamide diacetate)	Anti‐ageing (reduces wrinkles)	Acetylcholine receptor antagonist similar to the viper venom protein, waglerin‐1.^45^, ^51^

Abbreviations: Ac, acetyl; Cit, citrulline; Cu, copper; ECM, extracellular matrix; IL, interleukin; MITF, microphthalmia‐associated transcription factor; MMP, matrix metalloproteinase; Mn, manganese; Nrf2, nuclear factor erythroid 2‐related factor 2; Pal, palmitoyl; SNAP‐25, synaptosome‐associated protein (25 kDa); SNARE, soluble N‐ethylmaleimide‐sensitive factor activating protein receptor; TGF‐β, transforming growth factor beta; UV, ultraviolet.

Signal peptides or matrikines, are defined as peptides derived specifically from extracellular matrix (ECM) proteins such as collagen, elastin and fibronectin.^49^ Matrikines interact with specific receptors to stimulate ECM synthesis, repair and remodelling. These peptides also regulate the activity of certain key enzymes involved in the ageing process such as MMPs, collagenase, elastase, tyrosinase and hyaluronidase.^1^ Signal peptides are used in various topical skincare formulations for skin rejuvenation (improved skin firmness, elasticity, hydration and reduction of wrinkles).

Similarly to botulinum toxins, neurotransmitter inhibitor peptides prevent the release of acetylcholine, a neurotransmitter responsible for muscle contraction.^45^ The sequence of these peptides closely resembles that of the synaptic protein, SNAP‐25 (Synaptosome‐associated protein, 25 kDa), which mediates the signalling cascade for acetylcholine release.^44^, ^45^ Inhibition of this process relaxes the facial muscles thus preventing the formation of fine lines and wrinkles. These peptides are a safer alternative to the traditional Botulinum toxin treatments, with fewer potential side effects.

Although, peptides are widely used in topical formulations, the efficacy of this active ingredient is limited due to poor skin permeability and stability. Various structural modifications (e.g. conjugation, cyclisation, etc.) have been proposed to improve peptides' skin penetration and reduce degradation due to protease activity.^50^ Several techniques and drug delivery systems, including microneedling, radiofrequency, nanoparticles and liposomes, have been applied to improve transdermal peptide delivery.^50^ Readers are referred to a recent review by Ledwon and co‐workers for a more detailed discussion.^44^

## 5 ACIDS

### 5.1 Hyaluronic acid

The glycosaminoglycan, hyaluronic acid (also known as hyaluronan), is one of the main components of the ECM.^55^ One of hyaluronic acid's unique properties is its strong water retaining capacity, providing hydration and structural support to the epidermal and dermal layers of the skin.^55^ Hyaluronic acid is also involved in several aspects of the tissue repair process, including inflammatory cell activation, migration and proliferation of various cell types and angiogenesis.^55^ Hyaluronic acid's specific functions are dependent on its molecular size. Larger hyaluronic polymers exhibit anti‐inflammatory and anti‐angiogenic properties, while smaller hyaluronic fragments induce inflammation (via CD44 receptors and the receptor for hyaluronan‐mediated motility or RHAMM) and stimulate endothelial cell migration and proliferation for angiogenesis.^55^

With ageing, hyaluronic acid production decreases resulting in moisture loss (i.e. dry skin), reduced skin volume and elasticity, and formation of wrinkles and fine lines.^56^ Hyaluronic acid and its derivatives have been incorporated in various skin‐, hair‐ and nailcare products to moisturise and protect against oxidative stress (e.g. due to UV radiation) via its antioxidant properties.^57^ Sodium hyaluronate, a hyaluronic acid derivative, is often used in commercial products due to its stability and its smaller molecular structure which allows deeper skin penetration. It is used for hydration of various skin types (even oily and acne‐prone skin), tissue repair and to reduce dry eye syndrome.^58^, ^59^ Sodium hyaluronate has also demonstrated positive effects on the skin's microbiota, reducing pathogenic bacteria and stimulating the colonisation of beneficial bacteria.^59^

Several hyaluronic‐based injectable dermal fillers are available on the market to restore skin volume and reduce lines, folds and wrinkles.^60^ Although, the effects of hyaluronic acid fillers are temporary, these products are popular due to their safety and efficacy.^60^ Various cosmetics also incorporate hyaluronic acid to increase epidermal penetration of other active ingredients. These hyaluronic acid‐based drug delivery systems have demonstrated promising results in treatment of several inflammatory skin diseases such as atopic dermatitis, rosacea and psoriasis.^55^, ^57^, ^61^

### 5.2 Hydroxy acids

Hydroxy acids refer to a group of naturally occurring organic acids consisting primarily of four classes: α‐hydroxy acids (αHAs), β‐hydroxy acids (βHAs), polyhydroxy acids (PHAs) and bionic acids (BAs).^62^ Hydroxy acids are widely used as exfoliants in anti‐ageing products to stimulate epidermal cell turnover as well as collagen and elastin biosynthesis.^62^ This improves the overall texture, tone and pigmentation of the skin. At higher concentrations, hydroxy acids are used in the form of chemical peels to treat acne, photodamage, psoriasis and keratoses.^62^

αHΑs are hydrophilic acids such as glycolic acid, lactic acid, malic acid, citric acid, mandelic acid and phytic acid.^63^ PHAs (e.g. gluconolactone) and BAs (e.g. lactobionic acid) are structurally related to the αHA group; however, these acids demonstrate additional therapeutic advantages.^64^ Due to their larger structures, penetration of these acids is limited to the outer layers of the skin resulting in less irritation than αHAs.^64^ Thus PHAs and BAs are suitable for patients with sensitive skin and conditions such as rosacea and atopic dermatitis.^64^ These acids moisturise and strengthen the skin barrier and is often used to sooth irritated skin after certain cosmetic procedures.^64^ PHAs and BAs also have preventative anti‐ageing effects: inhibiting MMPs to prevent collagen degradation, and antioxidant properties to protect against photodamage.^64^ αHAs are generally used at concentrations <10%; however, higher concentrations are used for αHA peels.^63^ Adverse effects of traditional αHA formulations include erythema, pruritis, swelling, burning, dyspigmentation and at high concentrations increased photosensitivity.^63^ PHA and BA formulations are less likely to cause adverse effects, although skin irritation may still occur in some individuals.^64^

Salicylic acid is often classified as a βHA; however, its structure and function differ from traditional βHAs and is thus classified by some as a phenolic aromatic acid.^62^, ^65^ Due to its lipophilic nature, salicylic acid is miscible with the lipophilic stratum corneum and the sebaceous glands, making it ideal for treatment of acne in oily skin types.^65^ In addition to its exfoliating effect, salicylic acid also reduces sebum production, has anti‐inflammatory and antimicrobial properties, and has an anaesthetic effect which improves tolerability of salicylic acid peels.^65^ Salicylic acid concentrations in cosmetic products and chemical peels range between 0.5% and 50%.^65^ Topical salicylic acid formulations are generally well tolerated by all skin types; however, high concentrations may cause adverse effects such as dryness, erythema, crusting and dyspigmentation.^65^ Salicylism refers to systemic toxicity caused by cutaneous absorption of salicylic acid and may occur during high concentration salicylic acid peels applied to large areas of the body.^65^ The use of salicylic acid peels is contraindicated during pregnancy (due to its structural similarity to aspirin), for individuals with a salicylate allergy, active dermatitis and skin infection.^65^

### 5.3 Trichloroacetic acid

Trichloroacetic acid (TCA) is a monocarboxylic acid often used for superficial (10%–30% TCA) or medium‐depth (35%) chemical peels.^66^, ^67^ Its mechanism of action involves the coagulation of keratinocytes (i.e. keratocoagulation) and denaturation of proteins in the epidermal and dermal layers of the skin, thus stimulating skin re‐epithelialisation.^66^, ^67^ TCA peels are used to treat rhytides, actinic keratosis, photoaging, pigmentary dyschromia and acne scarring.^66^, ^67^ Low concentrations of TCA are generally safe with minimal side effects; however, hypopigmentation (especially in individuals with darker skin tones) and scarring can occur when higher concentrations are used.^66^

### 5.4 Ferulic acid

Ferulic acid is a phenolic acid with powerful antioxidant properties. This antioxidant scavenges free radicals, chelates catalytic metal ions, inhibits enzymes involved in ROS production, stabilises other antioxidants (e.g. L‐ascorbic acid and α‐tocopherol) and enhances the intracellular antioxidant systems.^68^ Ferulic acid's antioxidant activity also protects skin against UV‐induced damage, thus preventing photoaging.^68^, ^69^ In addition to its antioxidant activity, ferulic acid also demonstrates anti‐inflammatory, angiogenesis, skin‐lightening and wound healing effects.^68^, ^69^ Thus ferulic acid is used to treat signs of photoaging, hyperpigmentation and atopic dermatitis. Topical skincare products typically contain ferulic acid concentrations between 0.5% and 1%, and up to 12% in ferulic acid peels.^68^ Despite its many benefits and safety for most skin types, ferulic acid is readily oxidised and has limited solubility in water/oils/solvents used for cosmetic products.^69^

### 5.5 Kojic acid

Kojic acid, a fungal metabolite, is well‐known for its skin‐lightening effects and is used in various skincare products to address pigmentation disorders (e.g. post‐inflammatory hyperpigmentation, melasma, freckles and age spots).^70^ This effect is attributed to kojic acid's ability to inhibit tyrosinase activity, the main enzyme involved in melanogenesis.^70^ Kojic acid also demonstrates antioxidant, anti‐inflammatory, antimicrobial and antiproliferative activity.^70^, ^71^ Topical formulations typically contain kojic acid concentrations of up to 1%, which is considered safe and well‐tolerated for long‐term use.^70^, ^71^ The use of kojic acid products may result in contact dermatitis in individuals with sensitive skin.^71^

## 6 CANNABINOIDS

Cannabinoids refer to a group of structurally related compounds produced in the human body (endocannabinoids) and the *Cannabis Sativa* plant (phytocannabinoids), as well as synthetically produced cannabinoid derivatives.^72^ The biological effects of cannabinoids are mediated via multiple molecular targets including the endocannabinoid system.^73^, ^74^, ^75^ The endocannabinoid system is involved in numerous physiological processes and is distributed throughout the body, including the skin.^74^, ^75^ Cannabinoid signalling in the skin regulates homoeostasis, immune response, barrier function, cell proliferation and differentiation, melanogenesis and tissue repair.^74^ Therefore, dysregulation of cutaneous cannabinoid signalling has been implicated in several skin disorders including acne vulgaris, psoriasis, atopic dermatitis and cutaneous melanoma.^75^

The phytocannabinoid content of commercial products vary significantly based on cannabis strain, environmental factors affecting plant growth, part of the plant used (e.g. seeds, flowers, stems or leaves) and extraction method (e.g. CO_2_ extraction or steam distillation).^76^, ^77^ The two most abundant phytocannabinoids are tetrahydrocannabinol (THC, psychoactive cannabinoid) and cannabidiol (CBD).^74^ Over the counter cannabis products primarily contain CBD, either as an isolate (pure CBD) or as a mixture with other phytocannabinoids and plant compounds (e.g. terpenes, flavonoids and polyunsaturated fatty acids).^78^ Full spectrum cannabis products also contain THC (legal limit ≤0.3%), while broad spectrum products contain no or trace amounts of THC.^78^ Due to the various biological properties of phytocannabinoids (e.g. anti‐inflammatory, antioxidant, antiproliferative and antimicrobial), cannabis products have been proposed for treatment of several dermatological conditions.^74^ These conditions include acne, psoriasis, epidermolysis bullosa, androgenetic alopecia and melanoma.^74^ However, scientific evidence regarding their safety and efficacy are limited.

The effects of cannabinoids have mostly been studied in vitro and in animal models, with only a few clinical studies done thus far. Topical cannabinoid formulations seem to be well tolerated with less adverse effects (e.g. skin irritation and contact dermatitis) than oral administration.^74^ The following reviews provide an overview of cannabinoid research in different skin conditions, delivery systems for topical cannabinoid formulations and products available on the market.^72^, ^74^, ^75^, ^79^, ^80^

## 7 BOTULINUM TOXINS

Botulinum toxin is well known for its cosmetic use to reduce the appearance of facial lines and wrinkles. This neurotoxin inhibits the release of acetylcholine at the neuromuscular junction, resulting in temporary paralysis of the facial muscles.^81^ To date, the FDA has approved five botulinum toxin type A injections for cosmetic purposes. These include onabotulinumtoxinA (ONA), abobotulinumtoxinA (ABO), incobotulinumtoxinA (INCO), prabotulinumtoxinA (PRA) and daxibotulinumtoxinA (DAXI).^82^, ^83^ Although the active ingredient and mechanism of action are the same, these products differ in formulation, molecular potency and clinical effects.

Formulations differ in terms of the neurotoxin's molecular structure, diluents used in the manufacturing process, excipients and the method used for drying and finishing of the final product (Table 4). The molecular structure of ONA, ABO and PRA consists of a core neurotoxin (150 kDa) surrounded by several complexing proteins, while INCO and DAXI only consist of free toxin.^83^ It is hypothesised that these complexing proteins do not affect product efficacy, as the proteins dissociate from the core during reconstitution and the toxin is injected in its free form.^81^ While most formulations contain human serum albumin as excipient, the DAXI formulation is free from any human‐ and animal‐derived components.^84^ Instead DAXI neurotoxin is formulated using a synthetic stabilising peptide (RTP004) which is said to enhance neurotoxin binding to the presynaptic nerve terminal.^84^

**TABLE 4:** Molecular characteristics and approved aesthetic indications of commercial botulinum toxin type A formulations.^83^, ^84^

	ONA (Botox^®^, Vistabel^®^)	ABO (Dysport^®^, Azzalure^®^)	INCO (Xeomin^®^, Bocouture^®^)	PRA (Jeuveau™)	DAXI (Daxxify™)
Molecular weight (kDa)	900	Undisclosed by manufacturer^a^	150	900	150
Complexing proteins^b^	Yes	Yes	No	Yes	No
Excipients	Human serum albumin, Sodium chloride	Human serum albumin, Lactose	Human serum albumin, Sucrose	Human serum albumin, Sodium chloride	RTP004 peptide, Polysorbate‐20, Buffers, Sugar
Diluent	Saline	Gelatine phosphate buffer	Human serum albumin	Saline	Saline
Drying & finishing method	Vacuum‐dried	Lyophilised	Lyophilised	Vacuum‐dried	Lyophilised
FDA‐approved aesthetic indications	Moderate to severe forehead lines, lateral canthal lines and/or glabellar lines	Moderate to severe glabellar lines	Moderate to severe glabellar lines	Moderate to severe glabellar lines	Moderate to severe glabellar lines

^a^ Complex size is speculated to be around 500 kDa.^83^

^b^ Complexing proteins consist of several haemagglutinin proteins and a single non‐haemagglutinin protein.^85^

Abbreviations: ABO, abobotulinumtoxinA; DAXI, daxibotulinumtoxinA; INCO, incobotulinumtoxinA; ONA, onabotulinumtoxinA; PRA, prabotulinumtoxinA.

Clinical effects of botulinum toxin type A products may vary due to several factors including molecular potency, tissue distribution and patient specific response. Commercial products demonstrate different molecular potencies (i.e. the amount of free, active neurotoxin available for binding), which is affected by formulation and the manufacturing process.^81^, ^83^ Measurement of molecular potency is also proprietary to each manufacturer, thus preventing direct comparisons of potency between different botulinum toxin brands.^85^ The neurotoxin's tissue distribution can be affected by the injection technique, injection volume, depth of injection, the number of injection sites and the patient's muscle density.^81^, ^85^ Exact localisation of the neurotoxin to the target tissue is critical to achieve the desired clinical effect and prevent paralysis of adjacent muscles. Clinical effects can also vary between patients due to intrinsic factors (e.g. age, gender, muscle type and mass) which influence neurotoxin receptor density.^81^, ^83^ Fewer neurotoxin receptors will result in reduced neurotoxin binding and clinical efficacy.

Botulinum toxins' duration of action is generally between 3 and 4 months, although DAXI formulations may last between 6 and 9 months^84^ Common adverse effects associated with botulinum toxin injections include pain, erythema, oedema, hypoesthesia and bruising at the injection site.^86^, ^87^ Unwanted diffusion of the toxin to adjacent areas may result in facial weakness and ptosis.^87^ Rarely serious adverse effects may occur such as anaphylaxis, dysphagia and respiratory problems.

Botulinum toxin has also demonstrated potential in other areas of dermatology including treatment of rosacea, psoriasis, androgenetic alopecia and pathological scarring. Readers are referred to recent reviews on the subject.^86^, ^87^

## 8 ALLANTOIN

Allantoin is a natural compound primarily extracted from the comfrey plant (*Symphytum officinale*) or synthetically produced for cosmetic purposes.^88^ Allantoin is often used to soothe dry, irritated skin due to its anti‐inflammatory, keratolytic and moisturising (i.e. emollient) properties.^89^ In vivo studies have also demonstrated allantoin's potential in wound healing.^89^, ^90^ It is suggested that allantoin facilitates wound healing by regulating inflammation, removing necrotic tissue, protecting against infection (antimicrobial activity), and stimulating fibroblast proliferation, epithelialisation and ECM synthesis.^90^ Topical products usually contain allantoin concentrations between 0.1% and 2%, which is considered safe even for sensitive skin.^89^

## 9 UREA

Urea is an endogenous humectant primarily responsible for hydration of the stratum corneum.^91^ In addition, urea also enhances the skin's barrier function presumably by regulating gene expression involved in keratinocyte differentiation, and the biosynthesis of epidermal lipids and antimicrobial peptides.^92^ Topical formulations containing low concentrations of urea (<10%) are typically used for their moisturising effects in skin conditions such as xerosis (dry skin), ichthyosis, atopic dermatitis and psoriasis.^91^, ^93^, ^94^ Higher urea concentration formulations (10%–40%) also demonstrate keratolytic effects, removing the outer layers of the skin and stimulating new skin cell proliferation.^91^, ^93^, ^94^ This is especially beneficial for skin and nail conditions characterised by hyperkeratosis such as psoriasis, ichthyosis, dermatitis and onychomycosis.^94^ Urea has demonstrated efficacy as monotherapy and in combination therapies where it enhances the penetration of corticosteroids and antifungal drugs.^91^ Topical preparations are generally well‐tolerated; however, irritation, burning and erythema can occur at higher concentrations and sensitive skin areas (e.g. around the eyes and lips).^93^

## 10 CAFFEINE

Caffeine is a popular ingredient in many topical anti‐ageing, anti‐cellulite and anti‐hair loss products. Caffeine's anti‐ageing effects are attributed to its antioxidant properties as well as its ability to inhibit collagenase and elastase responsible for ECM degradation.^95^ The use of caffeine as a sunscreen adjuvant may also provide additional ultraviolet B (UVB) protection, thus reducing photoaging and the risk of developing skin cancer.^95^ Caffeine exerts several actions that stimulate lipolysis and may be beneficial for reducing cellulite.^96^ This includes the inhibition of adipocyte phosphodiesterase resulting in increased cyclic adenosine monophosphate (cAMP) levels which stimulates lipase activity.^96^ Caffeine may also act as a hair growth stimulant by inhibiting 5‐α‐reductase and subsequent conversion of testosterone to dihydrotestosterone, the hormone involved in hair loss (androgenic alopecia).^97^ Few adverse effects have been reported for topical caffeine formulations including erythema (due to increased blood circulation) and mild irritation.^98^

## 11 ZINC

Zinc is an essential trace mineral which serves as a co‐factor for numerous enzymes and transcription factors. Zinc and its derivatives are incorporated in various skin‐ and haircare products due to its antioxidant, anti‐inflammatory, antimicrobial and wound healing properties.^99^ Zinc‐containing skincare products soothes irritated skin and may be beneficial for treatment of inflammatory skin conditions such as acne, eczema, rosacea and psoriasis.^99^ Diaper rash creams and ointments often contain zinc oxide which forms a protective barrier against moisture, infection and irritation.^99^ Zinc oxide is also a popular ingredient in mineral‐based sunscreens due to its broad‐spectrum UV protection (UVA and UVB).^99^ Zinc pyrithione is an active ingredient in many shampoos as it demonstrates fungicidal activity against *Pit*yrosporum (Malassezia) which is associated with dandruff and seborrhoeic dermatitis.^99^ Some deodorants also contain zinc derivates which eliminate odours either by inhibiting bacterial growth (e.g. zinc oxide) or inhibiting bacterial enzymes responsible for odorant formation (e.g. zinc glycinate and ricinoleate).^99^ Zinc derivatives such as zinc propionate, caprylate and undecylenate are often used in footcare products due to their antifungal, antiseptic and deodorising properties.^99^ Zinc‐dependant enzymes (e.g. alkaline phosphatase and MMPs) and transcription factors (e.g. zinc finger regulatory proteins) are involved in all phases of the wound healing process.^100^, ^101^ Thus zinc paste bandages, stockings and other zinc‐impregnated dressings have been indicated for treatment of wounds of different aetiologies.^100^

## 12 CONCLUSION

Cosmeceutical ingredients such as vitamins, peptides, hyaluronic acid, hydroxy acids and cannabinoids have demonstrated potential therapeutic benefit for anti‐ageing and several skin disorders. As cosmeceutical products are not regulated by the FDA, many marketing claims have been made with regards to their safety and efficacy, without scientific validation. Evidence for these cosmeceuticals is often limited to in vitro research, with very few clinical studies currently available. There is thus an urgent need for high‐quality clinical studies to access the efficacy, safety and effects on the skin microbiota of cosmeceutical ingredients.

## CONFLICT OF INTEREST STATEMENT

Dr. Judey Pretorius is the founder and managing director of Biomedical Emporium.

## AUTHOR CONTRIBUTIONS

**Chantalle Crous**: Writing – original draft (lead). **Judey Pretorius**: Resources (lead); Writing – review & editing (equal). **Anél Petzer**: Writing – review & editing (equal).

## ETHICS STATEMENT

Not applicable.

## Data Availability

Data sharing not applicable to this article as no datasets were generated or analysed during the current study.
